# Seroprevalence and disease burden of acute hepatitis A in adult population in South Korea

**DOI:** 10.1371/journal.pone.0186257

**Published:** 2017-10-24

**Authors:** Jin Gu Yoon, Min Joo Choi, Jae Won Yoon, Ji Yun Noh, Joon Young Song, Hee Jin Cheong, Woo Joo Kim

**Affiliations:** Division of Infectious Diseases, Department of Internal Medicine, Korea University College of Medicine, Seoul, Korea; Chang Gung Memorial Hospital Kaohsiung Branch, TAIWAN

## Abstract

**Background:**

Adult seroprevalence of HAV is decreasing in developed countries including South Korea, due to general sanitation improvement. Although hepatitis A vaccination was introduced in South Korea more than 20 years ago, recent infection rates have not decreased. In this study, we investigate the seroprevalence of anti-HAV IgG, and estimate the national disease burden of acute hepatitis A in adult population.

**Methods:**

Seroprevalence data were collected from health promotion center of Korea University Guro Hospital, in Seoul, Korea from 2010 to 2014. Data from adults (≥20-years) being tested for anti-HAV IgG were included. In addition, epidemiological and clinical data of patients diagnosed with acute hepatitis A from 2009 to 2013, were collected from Korean Statistical Information Service (KOSIS) and the National Health Insurance Service (NHIS) database. Data were stratified and compared by age groups.

**Results:**

A total of 11,177 subjects were tested for anti-HAV IgG from 2010 to 2014. Age-related seroprevalence showed relatively low seropositivity in young adults. Incidence of acute hepatitis A was highest in 2009 and lowest in 2013. When categorized by age group, adults in their 20s and 30s had more HAV infections and related-admissions than older adults. However, ICU admission rate and average insurance-covered cost was high in older adults.

**Conclusion:**

The anti-HAV IgG seropositivity in Korean younger adult population was low while the incidence of acute hepatitis A was high, especially in the 20–39 aged. However, a substantial number of older adults were infected, and required more intensive procedures and incurred higher insurance-covered medical costs.

## Introduction

Hepatitis A virus (HAV) is a major cause of acute viral hepatitis, and is mainly transmitted by contaminated food and water. Most childhood HAV infections are asymptomatic or mildly symptomatic, without the need for medical care, however, severe symptoms or complications may occur in adults. Occasionally, fulminant hepatitis may develop, and liver transplantation may also be required in some such cases. In developed countries, acute hepatitis A infection in adult population is one of the major public health concerns.

During childhood, natural immunity against HAV can be acquired by acute infection, thereby producing anti-HAV immunoglobulin G (anti-HAV IgG). The seroprevalence rate of anti-HAV IgG varies in different regions and countries. In recent years, HAV seropositivity in young adults is decreasing as a result of improved general hygiene and economic status. In South Korea, HAV seropositivity was more than 80% in teenagers during 1970s, and had decreased to less than 20% in 2007, highlighting a rapid decline in immunity against HAV [1]. This low HAV seropositivity in young adults may induce an increase in incidence of symptomatic acute hepatitis A and consequently, increase the national health economic burden.

Despite the growing concern, there is limited data available on recent seroprevalence of anti-HAV IgG and acute hepatitis A disease burden in South Korea. The Korean Society of Infectious Disease guideline for adult immunization schedule recommends hepatitis A vaccination to young adults aged 19–29 years without testing anti-HAV IgG level, and to those aged 30–49 years after testing anti-HAV IgG level [2]. Nevertheless, high cost of the vaccine and low awareness of hepatitis A have been obstacles to proper vaccination and disease control. In South Korea, the hepatitis A vaccine has been available since 1997 [3], however, the seropositivity remains low in young adults, and the incidence of acute hepatitis A continues to increase [4]. Therefore, defining a suitable age group to examine anti-HAV IgG before vaccination, or recommending vaccination without antibody level examination are essential to establishing a comprehensive health care policy to counter HAV infections. In this study, we investigated anti-HAV IgG seroprevalence in adult population using the data from a health promotion center. Furthermore, national disease burden including insurance-covered cost of acute hepatitis A was studied using the National Health Insurance Service (NHIS) data.

## Methods

### HAV seroprevalence

The HAV seroprevalence data were collected from the health promotion center of Korea University Guro Hospital in Seoul, Korea, from 2010 to 2014. Adults aged over 19 years, who visited the center for medical examination and tested for anti-HAV IgG, were included in the study. Subjects were categorized into age groups as follows: 20–24 years, 25–29 years, 30–34 years, 35–39 years, 40–44 years, 45–49 years, 50–54 years, 55–59 years and 60-years or more. The whole process of data access and collection were confirmed as suitable by Institutional Review Board.

### Epidemiology and disease burden of hepatitis A

In South Korea, a patient diagnosed with acute hepatitis A should be reported to the community health center and recorded at the Korean Centers for Disease Control and Prevention, and the Korean Statistical Information Service (KOSIS). The NHIS provides national medical insurance under control of the Ministry of Health and Welfare. Almost the entire South Korean population is registered to the service and all NHI-covered healthcare costs are reported and assessed by Health Insurance Review and Assessment Service. Annual epidemiological data of acute hepatitis A were obtained from both KOSIS and NHIS databases. The NHIS database includes detailed patient information, such as date of admission, place of residence, gender, age, etc., unlike the KOSIS database. Also, the NHIS database includes all NHI-covered costs of admissions including medical procedures, surgeries, prescribed drugs, blood products, etc. The KOSIS database had only 3 years of relevant data (from 2011 to 2013), as the recording of hepatitis A incidences as KOSIS was launched only in 2011. From 2009 to 2013, adults aged 20–59 years and registered at the NHIS were included in this study. The study population was categorized by age, in 10-year intervals: 20–29 years, 30–39 years, 40–49 years and 50–59 years. In the NHIS data, cases of hepatitis A infection were identified using the Korean Standard Classification of Disease (KCD)-6 codes: B15.0 for hepatitis A with hepatic coma and B15.9 for hepatitis A without hepatic coma. Data collected for each patient included: duration of admission, admission to intensive care unit (ICU), mechanical ventilator use, continuous renal replacement therapy (CRRT) use, transfusion (fresh frozen plasma, platelet concentrate, or platelet plasmapheresis), liver transplantation, and cardiopulmonary resuscitation. Overall, NHI-covered direct medical costs including medical services and medication during hospitalization were also calculated for each age group.

### Statistics

Statistical analysis was performed using SPSS 15.0 software. Seropositivity and disease burden were compared among the various age groups. Difference of seropositivity between years and other categorical variables were analyzed using Chi-Square tests, while continuous variables were compared using ANOVA. A p-value < 0.05 was considered statistically significant.

### Ethics

This study is approved by the Institutional Review Board at the Korea University Guro Hospital in Seoul, Korea (KUGH15146-001). The informed consent was waived because the study is retrospective. The data were accessed securely and anonymously.

## Results

### HAV seroprevalence

A total of 47,289 adults visited the health promotion center between 2010 and 2014. A total of 11,177 subjects (23.6%) among the total 47,289, were tested for anti-HAV IgG levels; the year-wise distribution is as follows: 817 (9.1%) of 8,976 in 2010; 1,811 (19.0%) of 9,507 in 2011; 2,258 (24.2%) of 9.319 in 2012; 3,993 (39.7%) of 10,058 in 2013; and 2,298 (24.4%) of 9,429 in 2014.

**Fig 1:**
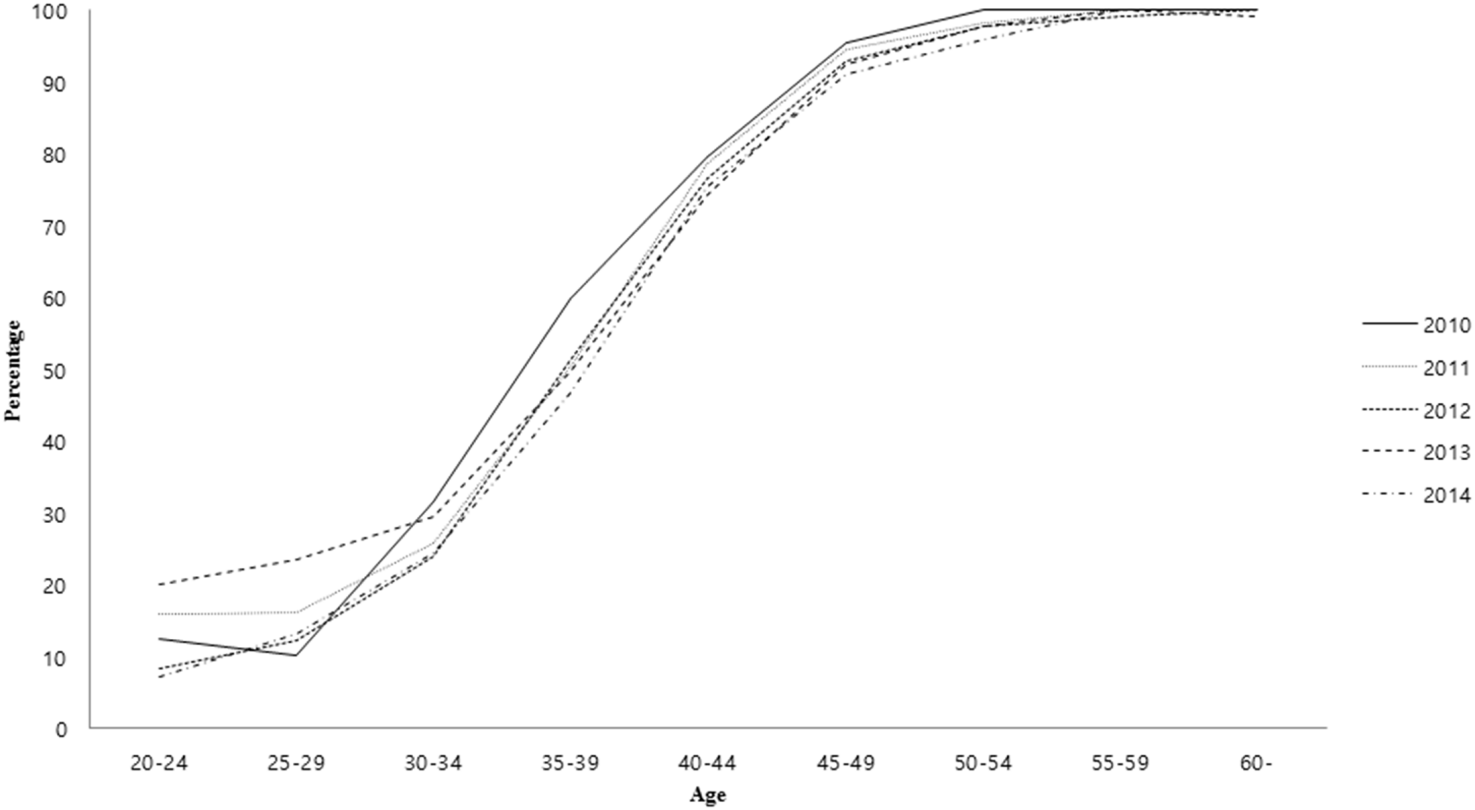
Age-stratified anti-HAV IgG seropositivity from 2010 to 2014.

A comparison of anti-HAV IgG seroprevalence across the age groups revealed that the HAV seroprevalence increased with age. Young age groups exhibited low HAV seropositivity (20–24 years, 12.7%; 25–29 years, 16.0%; 30–34 years, 26.7%). In addition, older age groups also showed moderate HAV seropositivity (35–39 years, 50.5%; 40–44 years, 76.0%). In contrast, more than 90% of the above 45-years-old population was HAV seropositive, with the age group-wise distribution as follows: 45–49 years, 92.6%; 50–54 years, 97.4%; 55–59 years, 99.8%; over 60-years-old, 99.7%). There was no significant difference between years in each age group (Table 1, Fig 1).

**Table 1:** Age-stratified anti-HAV IgG seropositivity from 2010 to 2014.

Age group	Years						
	2010	2011	2012	2013	2014	Total	P-value
20–24	1/8 (12.5%)	3/19 (15.8%)	1/12 (8.3%)	2/10 (20.0%)	1/14 (7.1%)	8/63 (12.7%)	.535
25–29	5/49 (10.2%)	9/56 (16.1%)	10/81 (12.3%)	20/85 (23.5%)	8/61 (13.1%)	53/332 (16.0%)	.150
30–34	25/79 (31.6%)	83/323 (25.7%)	67/279 (24.0%)	125/424 (29.5%)	49/202 (24.3%)	349/1307 (26.7%)	.447
35–39	111/186 (59.7%)	264/525 (50.3%)	333/651 (51.2%)	572/1150 (49.7%)	159/340 (46.8%)	1439/2852 (50.5%)	.091
40–44	151/190 (79.5%)	277/352 (78.7%)	347/453 (76.6%)	668/899 (74.3%)	370/490 (75.5%)	1813/2384 (76.0%)	.806
45–49	121/127 (95.3%)	244/258 (94.6%)	311/335 (92.8%)	583/631 (92.4%)	509/559 (91.1%)	1768/1910 (92.6%)	.516
50–54	120/120 (100.0%)	159/162 (98.1%)	252/258 (97.7%)	439/449 (97.8%)	388/405 (95.8%)	1358/1394 (97.4%)	.091
55–59	43/43 (100.0%)	68/68 (100.0%)	98/99 (99.0%)	216/216 (100.0%)	137/137 (100.0%)	562/563 (99.8%)	.320
60-	15/15 (100.0%)	48/48 (100.0%)	90/90 (100.0%)	128/129 (99.2%)	89/89 (100.0%)	370/371 (99.7%)	.758
Total	592/817	1155/1811	1509/2258	2753/3993	1710/2298		

### Epidemiology and disease burden of hepatitis A

**Fig 2:**
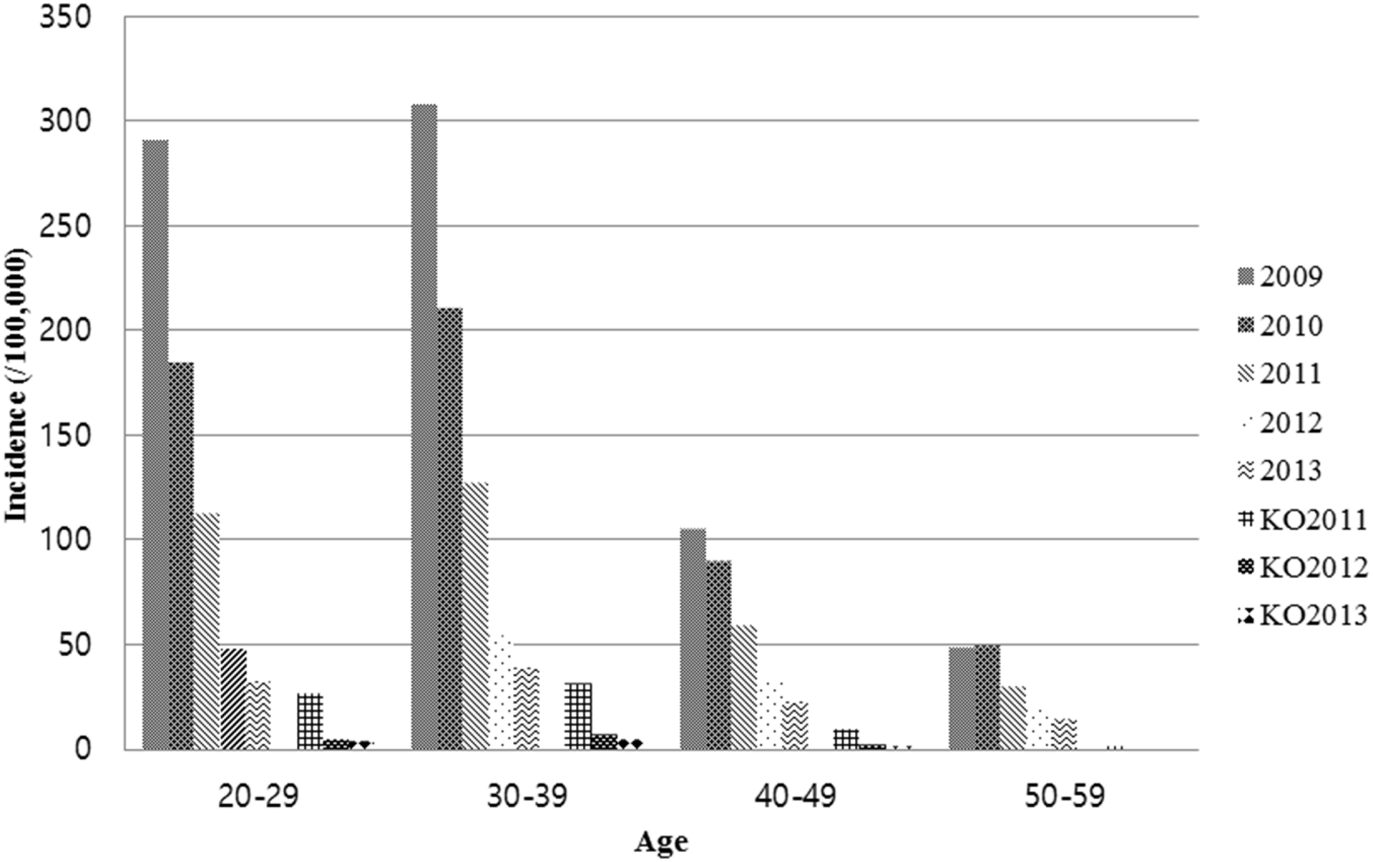
Incidence of acute hepatitis A by age group from 2009 to 2013 according to National Health Insurance data; KO2011~2013, Korean Statistical Information Service data from 2011 to 2013.

**Fig 3:**
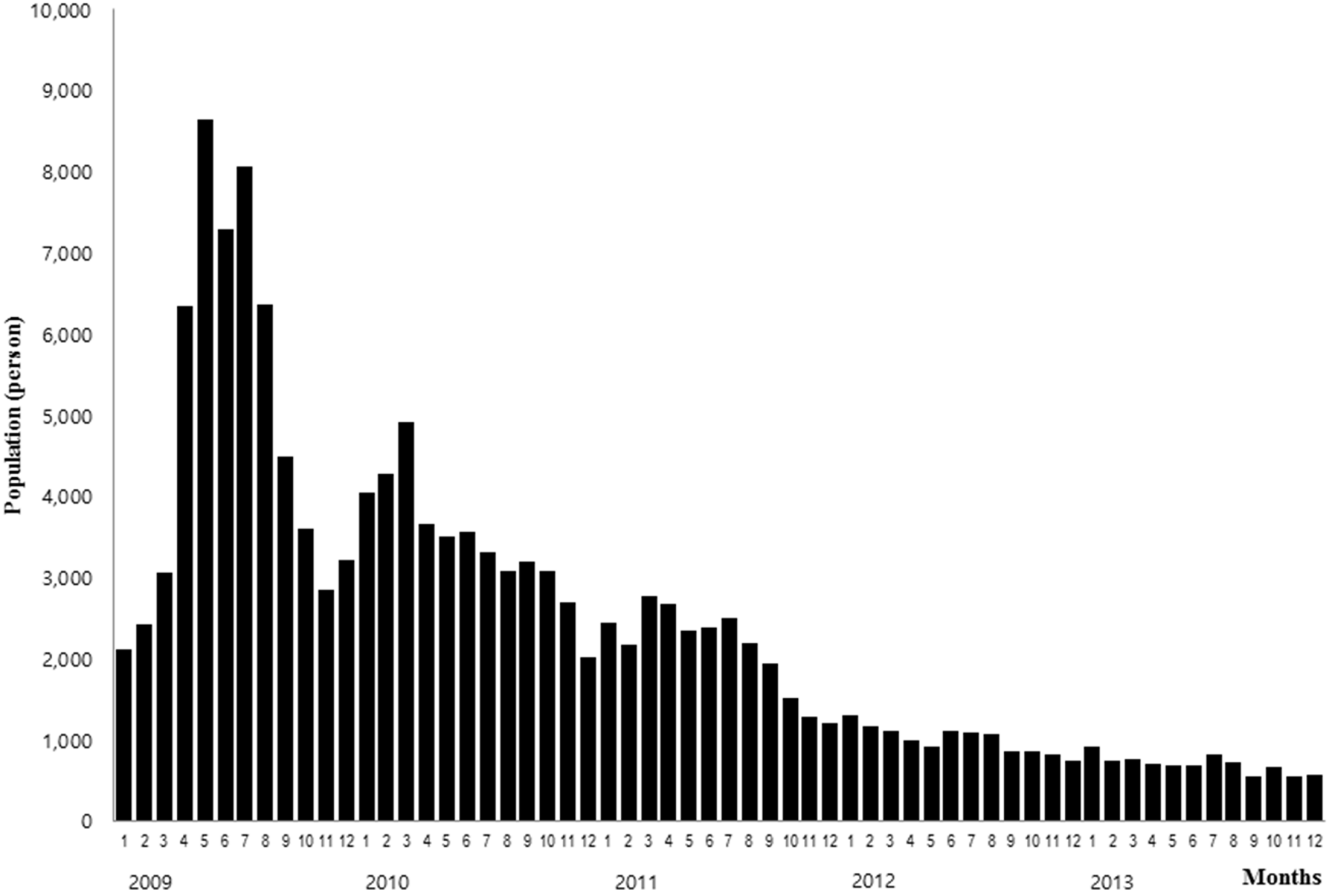
Monthly occurrence of acute hepatitis A from 2009 to 2013.

According to the NHIS data, the incidence of acute hepatitis A infection decreased continuously, from 2009 (N = 58,651, 192.8 cases per 100,000 population) to 2013 (N = 8,345, 26.7 cases per 100,000 population). In addition, the infection incidences were particularly high in the springs and summers (Figs 2 and 3, Table 2). Based on the classification of cases by region, the incidence was higher in the mid-western region of South Korea, including Seoul, Daejeon, Gwangju and Kyunggi. In contrast, the incidence was lower in the south-east region, including Busan, Daegu, Kyungbuk and Kyungnam.

**Table 2:** Age-related incidence of acute hepatitis A in adult aged 20–59 years.

	Year							
	2009	2010	2011		2012		2013	
	NHIS	NHIS	KOSIS	NHIS	KOSIS	NHIS	KOSIS	NHIS
Case number								
20–29 yrs	20,632	12,737	1,753	7,637	327	3,236	253	2,171
30–39 yrs	25,944	17,642	2,443	10,545	519	4,423	358	3,116
40–49 yrs	9,000	7,615	767	5,098	185	2,809	120	1,955
50–59 yrs	3,075	3,344	102	2,170	28	1,551	37	1,103
Total	58,651	41,338	5,065	25,450	1,059	12,019	768	8,345
Incidence by age (/100,000)								
20–29 yrs	291.3	184.8	26.6	112.8	5.0	48.4	3.8	32.6
30–39 yrs	308.5	211.4	31.3	127.6	6.7	54.0	4.6	38.6
40–49 yrs	105.1	90.0	9.3	59.2	2.3	32.5	1.5	22.3
50–59 yrs	48.3	49.2	1.6	29.8	0.4	20.5	0.6	14.1
Total incidence (/100,000)	192.8	135.1		82.3		38.7		26.7

Abbreviation: NHIS, National Health Insurance Service; KOSIS, Korean Statistical Information Service.

Hospital admission rate because of HAV infection was highest in the 30–39 years age group, with 17,138 cases (42.5 cases/100,000 population per year) and lowest in the 50–59 years age group, with 1,691 cases (4.3 cases/100,000 population per year). Duration of admission due to HAV infection was longer for subjects aged over 30 years’ (7.46 days in 20–29 years, 12.60 days in 30–39 years, 11.88 days in 40–49 years, 13.87 days in 50–59 years, p<0.0001). Furthermore, NHI-covered per capita medical cost was also higher with increasing age (people in 20s: 2,818,904 KRW, 30s: 3,701,382 KRW, 40s: 6,770,345 KRW, 50s: 9,711,832 KRW, p<0.0001). Total medical cost was highest in the 30–39 years age group. Case of mechanical ventilator application, CRRT, transfusion and liver transplantation were more frequent in the 30-39-years-old subjects in absolute case number, but the proportion of performing the above-mentioned procedures were higher in the older population. CPR rate indirectly measures the in-hospital mortality, and this was also higher in the older population: 4 in 46,413 (0.03%), 20–29 years; 3 in 61,670 (0.02%), 30–39 years; 4 in 26,477 (0.28%), 40–49 years; and 3 in 11,243 (0.18%), 50–59 years. All medical procedure rate were different significantly (p<0.01) (Table 3).

**Table 3:** Disease burden of acute hepatitis A from 2009 to 2013: Severity and NHI-covered medical cost stratified by age group.

	Age (years)				
	20–29 (n = 46,413)	30–39 (n = 61,670)	40–49 (n = 26,477)	50–59 (n = 11,243)	p-value
Admission (%)	12,915 (27.8)	17,138 (55.6)	5,544 (20.9)	1,691 (15.0)	
Duration of admission (day)(mean±SD)	7.46±10.60	12.60±12.6	11.88±16.2	13.87±18.15	<0.0001
ICU admission (%)	41 (0.31)	123 (0.72)	66 (1.19)	35 (2.07)	<0.01
Mechanical ventilator (%)	10 (0.08)	32 (0.19)	15 (0.27)	7 (0.41)	<0.01
CRRT (%)	3 (0.02)	12 (0.07)	8 (0.14)	3 (0.18)	<0.01
FFP (%)	130 (1.00)	203 (1.18)	99 (1.79)	19 (1.12)	<0.01
PC (%)	27 (0.21)	59 (0.34)	26 (0.47)	15 (0.89)	<0.01
Plasmapheresis (%)	4 (0.03)	8 (0.05)	0	0	<0.01
Liver transplantation (%)	4 (0.03)	22 (0.13)	8 (0.14)	2 (0.12)	<0.01
CPR (%)	4 (0.03)	3 (0.02)	4 (0.28)	3 (0.18)	<0.01
Medical cost per capita(KRW)(median,IQR)	928,947 (675,050–1,250,246)	966,420 (693,245–1,324,643)	1,013,379 (698,492–1,441,207)	1,023,831 (599,115–1,592,735)	
Medical cost per capita (KRW)(mean±SD)	2,818,904 ±7,561,130	3,701,382 ±9,489,901	6,770,345 ±15,266,028	9,711,832 ±18,409,706	<0.0001
Total medical cost(KRW)	130.8billion	228.3billion	179.3billion	109.2billion	

Abbreviation: SD, Standard deviation; ICU, Intensive care unit; CRRT, Continuous renal replacement therapy; FFP, Fresh frozen plasma; PC, Platelet concentration; CPR, Cardiopulmonary resuscitation; KRW, Korean Won; IQR, Interquartile range

## Discussion

HAV is the only member of the genus *Hepatovirus* in the *Picornaviridae* family. The viral particle, containing the single-stranded RNA genome, is fairly stable in the environment, and is transmitted via water and food contaminated with fecal matter [5]. Acute hepatitis A in adults has a more severe progression than in children, and may result in fulminant hepatitis, acute kidney injury, and even death in some cases. In developed countries including South Korea, hepatitis A infection during childhood is decreasing as a result of improved general hygiene [6]. Accordingly, adolescents and young adult population have lower seroprevalence of the protective antibody acquired by natural infection and hence more susceptible to acute hepatitis A infection in adult. Thus, investigating the current seroprevalence of anti-HAV IgG, and the disease burden of hepatitis A, will aid in the efforts toward infection control.

In South Korea, sporadic endemic hepatitis A outbreaks were reported since 1990, and the incidence increased strikingly after 2000, the year of designation of hepatitis A as a national notifiable infectious disease [1]. In spite of the HAV vaccine being available since 1997, the routine hepatitis A vaccination for children started just since 2015. Further, the rate of catch-up vaccination for young adults remains low due to high cost and low level of knowledge and awareness [3]. There is no large-scale, reliable study of hepatitis A vaccination rate in South Korea; however, several studies have reported vaccination rates to be 18.9% in adolescents, and 12.9–23.4% in college students [7–9]. In addition, the anti-HAV IgG seroprevalence in young adults is quite low in South Korea, especially for people in their 20s and 30s. Many Korean seroprevalence studies have demonstrated that only 1.0–18.8% of the 20-29-years-old, 26.0–48.4% of the 30-34-years-old, and 39.4–80.2% of the 35-39-years-old adults had the anti-HAV IgG antibodies [10–16]. Out study also demonstrated low seropositivity in people in their 20s (12.7% in 20-24-years-old, 16.0% in 25-29-years-old) and early 30s (26.7% in 30-34-years-old). Therefore, a substantial risk of acute hepatitis A outbreak among younger adult populations still exists, and may continue to grow for a while. To ensure cost-effectiveness, the Korean Society of Infectious Disease guideline recommends anti-HAV IgG check before vaccination for people in their 30s. However, active catch-up immunization without antibody check may be required, especially in early 30s. Epidemiologic and clinical data from recent studies display similar results; from 2007 to 2009, data from 21 tertiary hospitals showed 4,218 hepatitis A patients, mainly in their 30s. [17].

In our study, the incidence of hepatitis A varied among the different regions of Korea. A previous epidemiological study during 2011–2013, reported a similar prevalence of acute hepatitis A in the mid-western region, as we report here [18]. This suggests common infection source such as water supply system or other contaminated foods sources. The European hepatitis A outbreak in 2013 was believed to be associated with mixed frozen berries, raw shellfish, and traveling to/from endemic countries [19, 20]. Further studies with molecular analysis would be needed to investigate the cause of hepatitis A endemicity in South Korea.

A study by Shon et al. reported the estimated national economic burden of hepatitis A, B, and C per year, including the direct and indirect costs [21]. However, there is no study showing age-related difference of hepatitis A disease burden in South Korea. In this study, the overall number of patients and admission rate were higher in young adults; however, the individual ICU admission rates, mechanical ventilator use, CRRT use, liver transplantation rate, conduction of CPR, and total NHI-covered costs were higher in the older adults. This may be explained as the older adults may have more underlying illnesses or chronic diseases, which can contribute to more severe disease progression than in the young adults. Confounding factors such as age-related factors, or underlying illnesses, could not be considered in our study because patient’s past history from the NHI database was not reliable. Further studies with more reliable and detailed patient information would be needed to better analyze the disease burden. The NHI database also did not include accurate mortality data, and patient’s death had to be inferred from the CPR data. Higher mean costs, with large standard deviation than median costs indirectly express unbalanced distribution of medical cost per individuals due to various disease severity.

Our study had several limitations. First, a selection bias may exist, as the seroprevalence data were collected from only a single center and visited by almost healthy person with stable socioeconomic status. Therefore, the data may not reflect the entire population in Korea. Second, substantial omitted report, preliminary diagnosis, or misdiagnosis may obfuscate the true incidence of hepatitis A. The KOSIS data, reported by local physicians, showed a much lower HAV infection incidence compared to the NHIS data, which is automatically saved by inputting the diagnosis. It is difficult to determine the reliability of one dataset over the other, and more reasonable and readily accessible surveillance system for hepatitis A would be necessary. Third, each patient’s non-NHI-covered medical cost, as well as indirect costs such as loss of labor, was not considered.

In conclusion, hepatitis A seropositivity was considerably low, especially in the young adults (20-34-years old). Recent cases of acute hepatitis A are decreasing; however, new outbreaks may occur in this at-risk population. The incidence and admission rate of acute hepatitis A in young adults was higher, and associated with substantial disease burden. Therefore, hepatitis A immunization must promote not only routine vaccination for children, but also catch-up vaccination for young adults. Strengthened support and active public campaign towards young adult vaccination are essential in South Korea.
